# Clinical significance of immunohistochemical expression of DDR1 and *β*-catenin in colorectal carcinoma

**DOI:** 10.1186/s12957-023-03041-6

**Published:** 2023-06-05

**Authors:** Marwa Mohammed Dawoud, Marwa Salah, Asmaa Shams El Dein Mohamed

**Affiliations:** Department of Pathology, Faculty of Medicine, Menoufia University, Shebin El Kom, Egypt

**Keywords:** DDR1, *β*-*catenin*, Colorectal carcinoma, Immunohistochemical

## Abstract

**Background:**

Despite recent advances in therapy modalities of colorectal cancer (CRC), it is still the third cause of cancer-related deaths worldwide. Thus, the search for new target therapies became mandatory. DDR1 is a collagen receptor that has a suggested role in cellular proliferation, tumor invasion, and metastasis.

**Material and methods:**

Forty-eight cases of CRC, 20 of CR adenoma, and 8 cases of non-tumoral colonic tissue were subjected to immunohistochemistry by DDR1 and *β*-*catenin* antibodies. Results were compared among the different studied groups and correlated with clinicopathologic data and available survival data. Also, the expression of both proteins was compared versus each other. Results were compared among the 3 studied groups and correlated with clinicopathologic and survival data.

**Results:**

It revealed a stepwise increase of DDR1 expression among studied groups toward carcinoma (*P* = 0.006). DDR1 expression showed a direct association with stage D in the modified Dukes’ staging system (*P* = 0.013), higher-grade histologic types (*P* = 0.008), and lymph node invasion (*P* = 0.028) but inverse correlation with the presence of intratumoral inflammatory response (TIR) (*P* = 0.001). The shortest OS was associated with strong intensity of DDR1 (*P* = 0.012). The DDR1 and *β*-*catenin* expressions were significantly correlated (*P* = 0.028), and the combined expression of both was correlated with TNM staging (*P* = 0.017).

**Conclusion:**

DDR1 overexpression is a frequent feature in CRC and CR adenoma. DDR1 is a poor prognostic factor and a suppressor of the TIR. DDR1 and *β*-*catenin* seem to have a synergistic action.

**Supplementary Information:**

The online version contains supplementary material available at 10.1186/s12957-023-03041-6.

## Introduction

Globally, colorectal cancer (CRC) comes as the fourth most common cancer and the third cause of cancer-related deaths. It occurs in males more than females. Its incidence rises 3–4 times more in developed countries than in developing nations [1]. Genomic and epigenomic instability is a hallmark feature of colorectal carcinogenesis [2]. In CRC, 20% of patients have metastases at the time of diagnosis, and metastasis to either the liver, lung, brain, or peritoneum is present in about 90% of patients with stage 4 disease [3]. Metastasis is a multistep cascade with epithelial-mesenchymal transition (EMT) being the cornerstone of tumor invasion and metastasis [4].

Discoidin domain receptors (DDRs) are collagen receptors with tyrosine kinase activity with a suggested role in cellular proliferation, tumor invasion, and metastasis [5]. DDR1 was highly expressed in several tumors including the prostate [6], lung [7], breast [8] ovary [9], pancreas [10], liver [11], and stomach [12]. In CRC, DDR1 expression was recently observed to be associated with more stromal infiltration, upregulation of epithelial-mesenchymal transition genes, transforming growth factor-β (TGF-β) signaling, angiogenesis, matrix remodelling pathways, and complement-mediated inflammation [5]. On the other hand, a growing body of evidence suggests that DDRs promote apoptosis and suppress tumor progression in various human cancers [13]. The latter may be due to the preventive effect of DDR1 on cellular migration by promoting cell-cell adhesion by stabilizing E-cadherin [14]. Given previous well-established data on the essentiality of the E-cadherin/*β*-catenin complex to maintain the integrity of epithelial cell-cell contact [15], and the critical role of *β*-catenin signaling during the development of colorectal cancer [16], this study was designed to investigate DDR1’s roles in colorectal cancer and colorectal adenoma and its proposed correlation to *β*-catenin which are under-validated and still need further exploration. This could open a gate toward new combined modalities in CRC therapies, especially in view of the emerging recent data about the inhibition of DDR1 kinase activity with nilotinib by reducing the *β*-catenin pathway [17].

## Material and methods

This retrospective study was conducted on 76 colon specimens including 48 CRC cases, 20 cases of colorectal adenoma, and 8 control cases (nonneoplastic tissue adjacent to carcinomatous tissue). All cases were collected from the archive of the Pathology Department of Menoufia Faculty of Medicine between Jan 2018 and Dec and 2019 after approval of the ethical committee of Menoufia Faculty of Medicine. The clinicopathologic data were collected from the patients’ records after consenting the patients. The clinical data of all carcinoma cases included the following: age, gender, recurrence, and overall survival. Data about gross including the presence of polyps, gross perforation, gross description, and tumor multiplicity and tumor size. In addition, histopathologic data were evaluated and scored after examination of the hematoxylin and eosin (H&E) stained sections including histopathologic type and grade (according to WHO classification of colorectal tumors [18] pathological stage (according to TNM AJCC, 8th Edition [19] lymphovascular invasion (LVI) [20], perineural invasion (PNI), the host immune response (HIR) (peritumoral inflammatory reaction) [21], and tumor budding status and scoring [18] necrosis, mitosis, apoptosis, and lymph nodes metastases. Regarding survival analysis, follow-up time is calculated in months, and survival time was calculated from the date of surgery to either the date of death or the last follow-up of the patient.

With map creation, tissue microarray (TMA) was constructed using (Quick-Ray Tissue Microarray System, Beecher Instruments, Silver Spring, MD, USA). Four-micron thick sections of the TMA blocks were then cut and mounted on positively charged slides for immunohistochemical staining.

### Immunohistochemistry technique

Sections stained by automated immunostainer (Dako, Agilent Technologies Inc., Santa Clara, USA). For heat retrieval, citrate buffer was used by immunostainer. Slides were stained automatically by primary diluted antibodies of DDR1 (rabbit polyclonal antibody, Santa Cruz Biotechnology, diluted at 1/100) and *β*-catenin (mouse monoclonal [5H10] antibody, Abcam, concentration of 1 µg/ml). Positive controls were desmoid tumor for *β*-catenin. Negative controls were made by substituting the primary antibodies with nonimmune serum.

### Interpretation of immunostaining results

Immuno-stained sections were scored by 2 pathologists independently and blindfolded from clinicopathologic data. DDR1 was evaluated in all studied cases (carcinoma, adenoma, and control cases). Interpretation of immunostaining included evaluation of expression (positive expression should be considered if any cytoplasmic or membranous brownish staining of cells) [22] localization (cytoplasmic or membranous), intensity (mild, moderate, and strong), extent (% percent of positivity), and calculation of H-score (calculated based on a linear combination of the percentage of weakly stained cytoplasm/nucleus (WSN)), the percentage of moderately stained cytoplasm/nucleus (MSN), and the percentage of strongly stained cytoplasm/nucleus (SSN) according to the equation: *H-score = 1 × WSN + 2 × MSN + 3 × SSN*. The final score has a numerical value range from 1 to 300 [23]. *β*-catenin was evaluated in carcinoma and adenoma cases. Interpretation of immunostaining included evaluation of expression (positive expression should be considered if any brownish staining of cells), localization (cytoplasmic, membranous, nuclear [24] intensity), extent (%), and calculation of H-score.

### Statistical analysis

Data were statistically analyzed using a Statistical Package for the Social Sciences (SPSS) program for windows, version 20, SPSS Inc., Chicago, IL, USA. Different types of statistics were used including descriptive statistics (mean and standard deviation (SD); percentage (%); median, range; Kolmogorov-Smirnov test) and analytic statistics (Mann-Whitney (*U*)-test; Kruskal-Wallis (K) test; Spearman’s rho (r) test; chi-square (*Χ*^2^) test; Fisher exact (FE) test; post hoc analysis of one-way ANOVA test; the unpaired (two-sample) Student’s (*t*)-test). Survival analysis included using Kaplan-Meier survival curves, hazard function curves, and multivariate Cox-regression analysis for detection of the most independent prognostic factor affecting overall survival. Differences were considered to be statistically significant when (*P* ≤ 0.05), highly significant when (*P* < 0.01), and near significant when (*P* < 0.08).

## Results

This study was conducted on 76 cases including 48 cases of CRC, 20 of adenoma, and 8 cases of non-tumoral colonic tissue. In terms of adenoma, 65% were low grade, 70% were tubulovillous, and their age ranged between 36 and 68 years with mean ± SD (57.9 ± 8.8). Carcinoma cases included 27 females and 21 males with an age range between 29 and 76 years and a mean ± SD (54.4 ± 12.7). The clinicopathologic features of carcinoma cases are summarized in Table 1.

**Table 1:** The clinicopathologic features of carcinoma cases (*n* = 48)

		**No. (%)**
**Presence of polyps**		1 (2.1%)
**Adjacent adenoma**		4 (8.3%)
**Multiple tumors**		2 (4.2%)
**Gross description**	Infiltrating	15 (31.3%)
	Fungating	24 (50%)
	Ulcerating	9 (18.8%)
**Gross perforation**		9 (18.8%)
**Grade**	Low & intermediate	39 (81.3%)
	High	9 (18.8%)
**T stage**	T2	7 (14.6%)
	T3	31 (64.6%)
	T4	10 (20.8%)
**N stage**	N0	28 (58.3%)
	N1	12 (25.0%)
	N2	8 (16.7%)
**M stage**	M0	36 (75%)
	M1	12 (25%)
**Modified Duke’s staging**	B	25 (52.1%)
	C	18 (37.5%)
	D	5 (10.4%)
**Combined Dukes**	B, C	43 (89.6%)
	D	5 (10.4%)
**Necrosis**		13 (27.1%)
**Apoptosis (*n* = 21)**	Mean ± SD	25.6 ± 22.1
	Median (min.–max.)	20 (2–90)
**Mitosis**	Mean ± SD	9.4 ± 6.4
	Median (min.–max.)	8.5 (1–28)
**Vascular invasion**		15 (31.3%)
**Perineural invasion**		11 (22.9%)
**Lymph node harvest**	Mean ± SD	14.6 ± 9.2
	Median (min.–max.)	12.5 (2–45)
**Lymph node metastasis [N_ combined]**	N0	41 (85.4%)
	N1, 2	7 (14.6%)
**Type of tumor/variant (*n* = 47)**	Signet	3 (6.4%)
	Adenocarcinoma	37 (78.7%)
	Mucinous	3 (6.4%)
	Adenocarcinoma with mucinous differentiation	4 (8.5%)
**Inflammatory response**		40 (85.1%)
**Tumor. budding**	Low (< 5)	22 (45.8%)
	Intermediate (5–9)	20 (41.7%)
	High (≥ 10)	6 (12.5%)
**Recurrence**		9 (18.8%)
**Death**		24 (50.0%)
**Overall survival (*n* = 45)**	Mean ± SD	18.76 ±11.98
	Median (min.–max.)	17 (1-50)

### Results of immunostaining

**Fig. 1:**
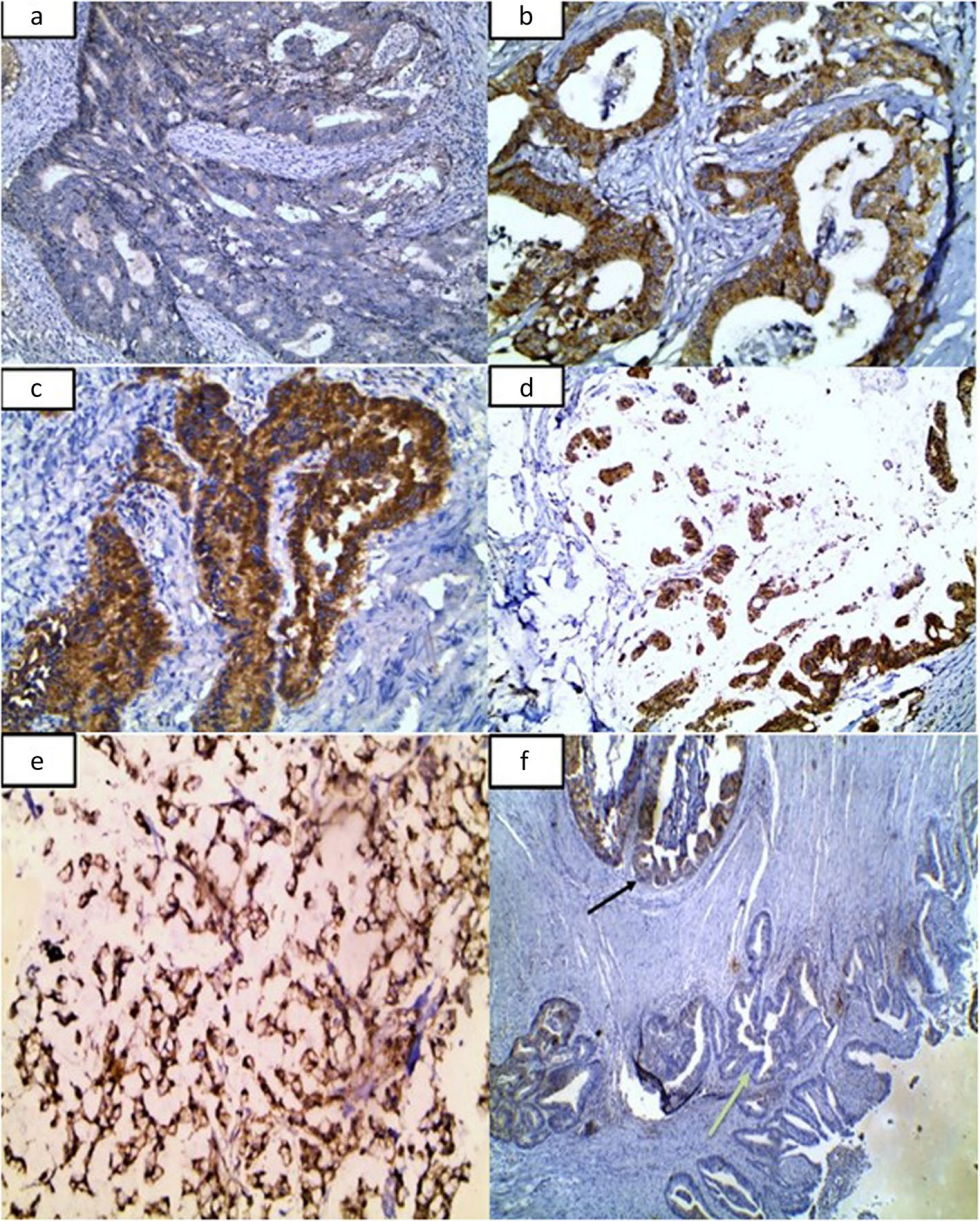
Sections of CRC immunostained by DDR1. **a** Mild scattered positive cytoplasmic expression of DDR1 in a case of moderately differentiated CRC (×200). **b** Moderate expression of DDR1 in a case of moderately differentiated CRC (×200). **c** Strong expression of DDR1 in a case of moderately differentiated CRC (×400). **d** Strong expression of DDR1 in a case of mucinous CRC (×200). **e** Strong expression of DDR1 in a case of signet ring CRC (×200). **f** Mild expression of DDR1 in a malignant acinus (black arrow) with overlying adjacent showing negative expression (green arrow) (×100)

Regarding the expression of DDR1, there was a significant association between DDR1 expression and carcinoma (*P* = 0.0056) where all cases were positive, and most of them exhibited moderate and strong expression (57.2%). Additionally, 95% of almost all adenoma cases were positive but with mild to moderate intensity. Regarding intracellular localization, most adjacent normal tissue exhibited cytomembremous localization (66.7%), while most carcinoma (77.1%) and all adenoma cases showed cytoplasmic expression. Comparison between the 3 studied groups showed a stepwise increase of H-score of DDR1 expression from adjacent normal (mean ± SD = 20 ± 8.9) to the carcinoma group (137.4 ± 96.9) (Table 2, Fig. 1).

**Fig. 2:**
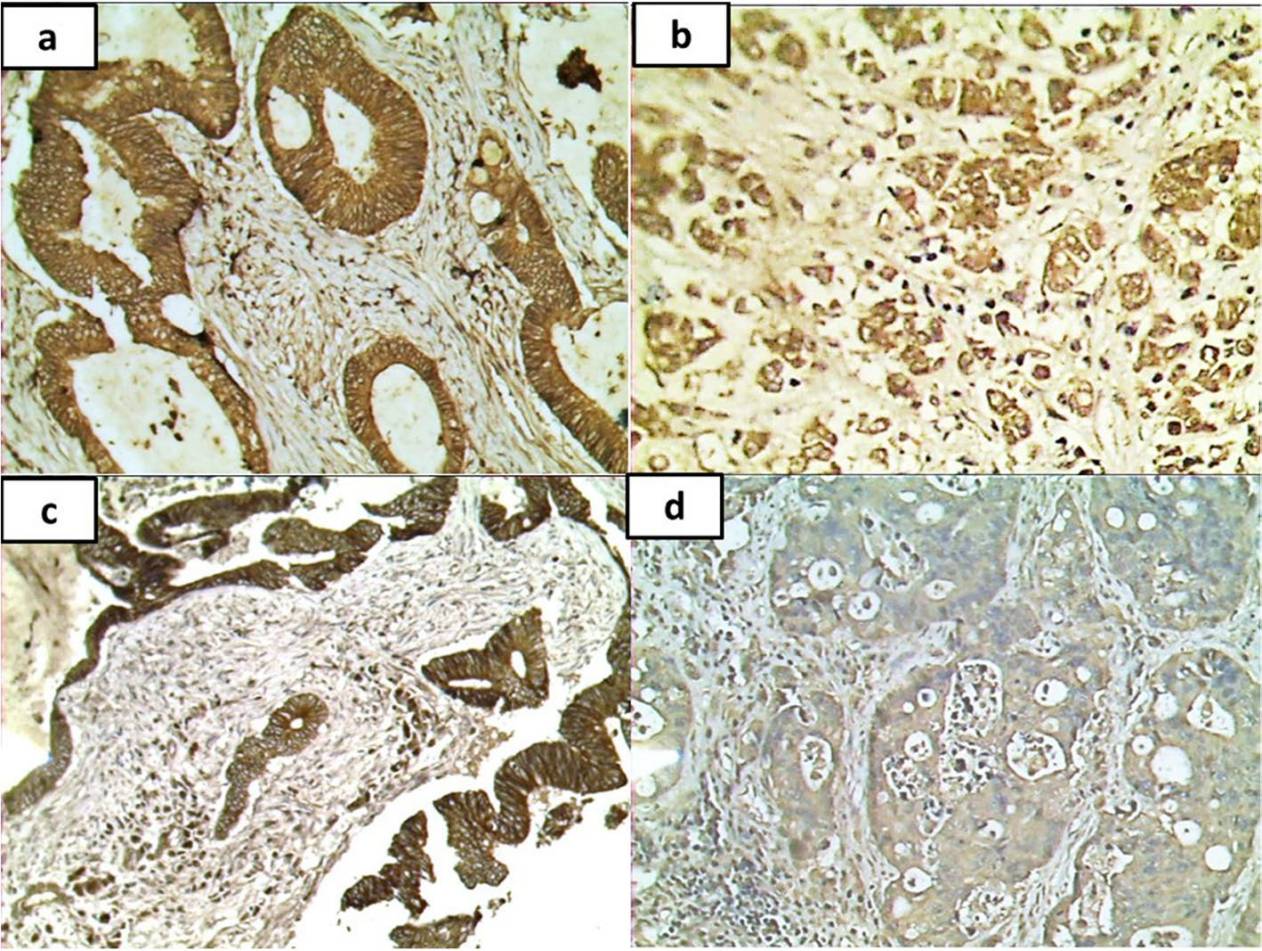
Sections of CRC immunostained by *β*-catenin. **a** Case of well/moderately differentiated adenocarcinoma, NOS exhibiting strong membranocytoplasmic expression in malignant glands (×200). **b** A case of signet ring exhibiting moderate cytoplasmic expression in malignant cells (×400). **c** A case of mucoid carcinoma exhibiting strong membranocytoplasmic expression in malignant glands (×200). **d** A case of well/moderately differentiated adenocarcinoma, NOS exhibiting negative expression in malignant glands (×200)

There was no significant difference in the comparison between adenoma and carcinoma regarding the expression of *β*-catenin. However, the expression tends to be significantly more in the adenoma group (90% versus 68.8%) (*P* = 0.06) (Table 2, Fig. 2).

**Table 2:** Comparison between adjacent normal, adenoma, and carcinoma cases according to DDR1 and *β*-catenin immunostaining (*n* = 76)

	**Adjacent normal (*n* = 8)**	**Adenoma (*n* = 20)**	**Carcinoma (*n* = 48)**	***p***_**1**_	***p***_**2**_	***p***_**3**_
**EpithelialDDR1 **						
**Intensity**				*P* = 0.0056^a^		
Negative	2 (25%)	1 (5.0%)	0 (0%)	MH 0.035^a^	MH 0.046^a^	MH 0.032^a^
Mild	2 (25%)	11 (55%)	22 (45.8%)			
Moderate	2 (25%)	8 (40%)	13 (27.1%)			
Strong	2 (25%)	0 (0.0%)	13 (27.1%)			
**Localization**	***n* = 6**	***n* = 19**	***n* = 48**	*MH* < 0.0001^a^	*MH* 0.739	NA
C	1 (16.7%)	19 (100%)	37 (77.1%)			
M	1 (16.7%)	0 (0%)	0 (0%)			
MC	4 (66.7%)	0 (0%)	11 (22.9%)			
**H-score**	***n* = 6**	***n* = 19**	***n* = 48**			
Mean ± SD	20 ± 8.9	81.5 ± 42.2	137.4 ± 96.9	*U* 0.001^a^	*Z* 0.027^a^	*U* 0.042^a^
Median (min.–max.)	20 (10–30)	80 (10–160)	140 (10–300)			
**Extent (%)**	***n* = 6**	***n* = 19**	***n* = 48**			
Mean ± SD.	10 ± 0		64.1 ± 32.8	NA	*Z* 0.024*	NA
Median (min.–max.)	10 (10-10)	NA	80 (10–100)			
***Epithelial β*-catenin**						
**Expression**						
Negative		2 (10%)	15 (31.3%)			*Χ*^2^ 0.06
Positive		18 (90%)	33 (68.8%)			
**Intensity**		***n* = 18**	***n* = 33**			
Mild		8 (44.4%)	15 (45.5%)			*Χ*^2^ 1.0
Moderate		8 (44.4%)	13 (39.4%)			
Strong		2 (11.1%)	5 (15.2%)			
**Localization**		***n* = 18**	***n* = 33**			
C		18 (100%)	25 (75.8%)			*Χ*^2^ 0.103
NC		0 (0%)	3 (9.4%)			
MC		0 (0%)	5 (15.6%)			
**H-score**		***n* = 18**	***n* = 33**			
Mean ± SD		113.3 ± 73.8	126.7 ± 77.24			*U* 0.422
Median (min.–max.)		130 (20-240)	160 (20-270)			
**Extent/percent (%)**		***n* = 18**	***n* = 33**			
Mean ± SD		61.7 ± 25.5	68.8 ± 23.3			*U* 0.194
Median (min.–max.)		70 (20–90)	80 (20–90)			

*C* Cytoplasmic, *NC* Nucleocytoplasmic *MC* Membrano-cytoplasmic, *Χ*^2^, chi-square test. *MC*, Monte Carlo. *MH*, marginal homogeneity test. *U* Mann-Whitney test. *Z* Wilcoxon signed-rank test. *P p*-value for comparing among the 3 studied group. *p*_1_, *p*-value for comparing between adjacent normal and adenoma. *p*_2_*p*-value for comparing between adjacent normal and carcinoma. *p*_3_, *p*-value for comparing between adenoma and carcinoma. ^a^Statistically significant at ≤ 0.05

### Relation between DDR1 expression in carcinoma and clinicopathological parameters

When the intensity of DDR1 expression in carcinoma was correlated with clinicopathological parameters, it was significantly associated with stage D in the modified Dukes’ staging system (*P* = 0.013). Additionally, strong DDR1 expression was significantly related to higher-grade types of CRC (mucinous and signet ring) (*P* = 0.008), and lymph node invasion (*P* = 0.028), and showed near significant association with metastasis (*P* = 0.08). In contrast, the presence of an intratumoral inflammatory response was inversely correlated with the intensity of DDR1 expression (*P* = 0.001).

Regarding overall survival (OS), which was available for 45 cases, the shortest OS was significantly associated with strong intensity of DDR1 (*P* = 0.012).

Concerning the relation between the expression intensity of DDR1 and *β*-catenin, there was a significant correlation (*P* = 0.025) (Table 3).

**Table 3:** Relation between the intensity of DDR1 and different parameters in carcinoma cases (*n* = 48)

		**Intensity of DDR1**			**Test of sig.**	***p***
		**Mild (*n* = 22)**	**Moderate (*n* = 13)**	**Strong (*n* = 13)**		
**Sex**	Male	11 (50%)	5 (38.5%)	5 (38.5%)	*Χ*^2^ = 0.645	0.724
	Female	11 (50%)	8 (61.5%)	8 (61.5%)		
**Age (years)**	Mean ± SD	53 ± 13.8	52.7 ± 11.2	58.5 ± 12.2	*F* = 0.920	0.394
	Median (min.–max.)	58.5 (29–75)	55 (30–70)	60 (34 – 76)		
**Presence of polyps**	Absent	21 (95.5%)	13 (100%)	12 (92.3%)	*Χ*^2^ = 1.160	^MC^*p* = 1.000
	Present	1 (4.5%)	0 (0%)	1 (7.7%)		
**Adenoma**	Absent	21 (95.5%)	13 (100%)	10 (76.9%)	*Χ*^2^ = 3.981	^MC^*p* = 0.148
	Present	1 (4.5%)	0 (0%)	3 (23.1%)		
**Multiple tumors**	Absent	22 (100%)	13 (100%)	11 (84.6%)	*Χ*^2^ = 3.759	^MC^*p* = 0.138
	Present	0 (0%)	0 (0%)	2 (15.4%)		
**Gross description**	Infiltrating	8 (36.4%)	3 (23.1%)	4 (30.8%)	*Χ*^2^ = 3.792	^MC^*p* = 0.448
	Fungating	8 (36.4%)	9 (69.2%)	7 (53.8%)		
	Ulcerating	6 (27.3%)	1 (7.7%)	2 (15.4%)		
**Gross perforation**	0	17 (77.3%)	11 (84.6%)	11 (84.6%)	*Χ*^2^ = 0.434	^MC^*p* = 0.804
	1	5 (22.7%)	2 (15.4%)	2 (15.4%)		
**Grade**	Low & intermediate	17 (77.3%)	11 (84.6%)	11 (84.6%)	*Χ*^2^ = 0.434	^MC^*p* = 0.808
	High	5 (22.7%)	2 (15.4%)	2 (15.4%)		
**T stage**	T2	2 (9.1%)	3 (23.1%)	2 (15.4%)	*Χ*^2^ = 1.682	^MC^*p* = 0.860
	T3	15 (68.2%)	8 (61.5%)	8 (61.5%)		
	T4	5 (22.7%)	2 (15.4%)	3 (23.1%)		
**N stage**	N0	10 (45.5%)	10 (76.9%)	8 (61.5%)	*Χ*^2^ = 4.165	^MC^*p* = 0.417
	N1	8 (36.4%)	1 (7.7%)	3 (23.1%)		
	N2	4 (18.2%)	2 (15.4%)	2 (15.4%)		
**M stage**	M0	17 (77.3%)	10 (76.9%)	9 (69.2%)	*Χ*^2^ = 0.443	^MC^*p* = 0.914
	M1	5 (22.7%)	3 (23.1%)	4 (30.8%)		
**Modified Dukes’ staging**	B	10 (45.5%)	9 (69.2%)	6 (46.2%)	*Χ*^2^ = 10.031*	^MC^*p* = 0.024^*^
	C	12 (54.5%)	3 (23.1%)	3 (23.1%)		
	D	0 (0%)	1 (7.7%)	4 (30.8%)		
**Combined Dukes’ staging**	B, C	22 (100%)	12 (92.3%)	9 (69.2%)	*Χ*^2^ = 7.150*	^MC^*p* = 0.013^*^
	D	0 (0%)	1 (7.7%)	4 (30.8%)		
**Necrosis**	Absent	16 (72.7%)	9 (69.2%)	10 (76.9%)	*Χ*^2^ = 0.293	^MC^*p* = 1.000
	Present	6 (27.3%)	4 (30.8%)	3 (23.1%)		
**Apoptosis (*n* = 21)**	Mean ± SD	26.3 ± 13.3	23.3 ± 23.1	26.1 ± 25.1	*H* = 0.181	0.914
	Median (min.–max.)	25 (14–41)	17.5 (2–56)	20 (2–90)		
**Mitosis**	Mean ± SD	8.8 ± 5.6	9.4 ± 6.5	10.4 ± 7.7	*H* = 0.159	0.924
	Median (min.–max.)	8 (1–22)	8 (2–28)	10 (2–26)		
		***n* = 22**	***n* = 13**	***n* = 13**		
**Vascular invasion**	Absent	18 (81.81%)	11 (84.61%)	4 (30.76%)	*Χ*^2^ = 12.00*	^MC^*p* = 0.095
	Present	4 (18.18%)	2 (15.38%)	9 (69.23%)		
**Lymph node harvest**	Mean ± SD	13.5 ± 7.9	15.5 ± 8.1	15.5 ± 12.2	*H* = 0.782	0.676
	Median (min.–max.)	11.5 (2–29)	14 (3–28)	13 (3–45)		
**Lymph node metastasis [N_ combined]**	N0	21 (95.5%)	12 (92.3%)	8 (61.5%)	*Χ*^2^ = 6.737*	^MC^*p* = 0.028*
	N1, 2	1 (4.5%)	1 (7.7%)	5 (38.5%)		
**Type of tumor/variant**	Signet	0 (0%)	0 (0%)	3 (%)	*Χ*^2^ = 13.776*	^MC^*p* = 0.0080*
	Adenocarcinoma	22 (100%)	12 (%)	7 (%)		
	Mucinous Adenocarcinoma with mucinous differentiation	0 (0%) 0 (0%)	1 (%) 0 (0%)	2 (%) 1 (%)		
**Inflammatory response (*n*** **= 47)**	Absent	0 (0%)	1 (7.7%)	6 (50.0%)	*Χ*^2^ = 13.291*	^MC^*p* < 0.001*
	Present	22 (100%)	12 (92.3%)	6 (50%)		
**Tumor budding**	Low (< 5)	10 (45.5%)	4 (30.8%)	8 (61.5%)	*Χ*^2^ = 4.195	^MC^*p* = 0.395
	Intermediate (5–9)	8 (36.4%)	7 (53.8%)	5 (38.5%)		
	High (≥ 10)	4 (18.2%)	2 (15.4%)	0 (0%)		
**Recurrence**	No	18 (81.8%)	10 (76.9%)	11 (84.6%)	*Χ*^2^ = 0.398	^MC^*p* = 1.000
	Yes	4 (18.2%)	3 (23.1%)	2 (15.4%)		
**Death**	No	10 (45.5%)	6 (46.2%)	8 (61.5%)	*Χ*^2^ = 0.951	0.622
	Yes	12 (54.5%)	7 (53.8%)	5 (38.5%)		
**Overall survival (*n* = 45)**	Mean ± SD	17.2 ± 11.5	26.8 ± 13.1	13.7 ± 7.8	*H* = 8.398*	0.012*
	Median (min.–max.)	15 (1–50)	27.5 (2–46)	14 (2–27)		
**Intensity of Epithelial *β*-catenin**	Negative	4 (18.2%)	4 (30.8%)	7 (53.8%)	*Χ*^2^ = 13.109*	^MC^*p* = 0.025*
	Mild	10 (45.5%)	4 (30.8%)	1 (7.7%)		
	Moderate	8 (36.4%)	2 (15.4%)	3 (23.1%)		
	Strong	0 (0%)	3 (23.1%)	2 (15.4%)		

*Χ*^2^ chi-square test. *MC*, Monte Carlo. *H* H for Kruskal-Wallis test. *p P*-value for comparing the intensity of DDR1 categories. *Statistically significant at *P* ≤ 0.05

When the H-score, the extent/percent of expression, and intracellular localization were correlated to the clinicopathological parameter, the H-score and percent of expression exhibited a significant direct correlation to Dukes’ stage (*P* = 0.004, *P* = 0.018, respectively), lymph node invasion (*P* = 0.001, *P* = 0.001, respectively), and high-grade histologic types (*P* = 0.02), and a significant inverse correlation to the intratumoral inflammatory response (*P* = 0.001, *P* = 0.004, respectively). Additionally, the correlation between DDR1 H-score and *β*-catenin H-score and between DDR1 percent of expression and metastases was nearly significant (*r* = 0.307, *P* = 0.08, *U* = 141.50, *P* = 0.07).

### Relation between β-catenin expression in carcinoma and clinicopathological parameters

When the H-score of *β****-****catenin* expression in carcinoma was correlated with clinicopathological parameters, it showed a significant direct association with the D stage of Duckes’ staging system (*P* = 0.025), near-significant association with the tumor (T) stage (*P* = 0.06), but inversely correlated to the intratumoral inflammatory response (*P* < 0.001).

### Relation between combined expression of DDR1 and β-catenin in carcinoma and clinicopathological parameters

**Fig. 3:**
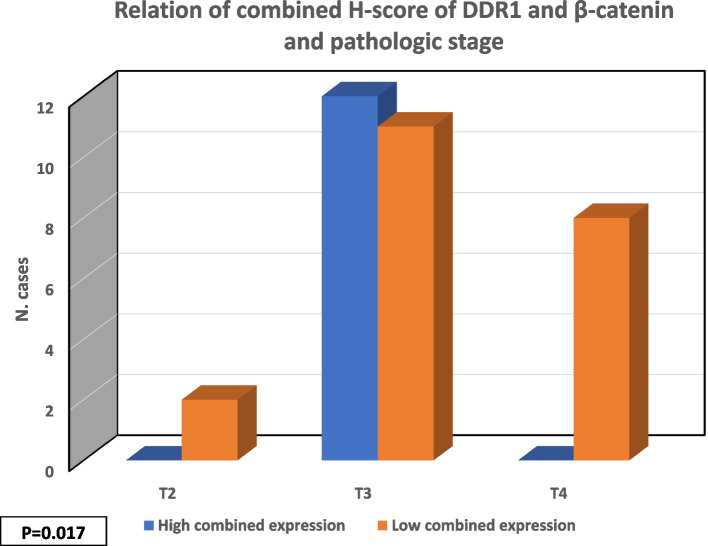
Significant correlation of combined H-score of DDR1 and *β*-catenin and TNM stage (*P* = 0.017)

When the H-score of DDR1 and *β***-***catenin* expression in carcinoma was correlated with clinicopathological parameters, it showed a significant association with the tumor (T) stage (*P* = 0.017) (Fig. 3). However, testing their possible impact on the overall survival in studied CRC cases by low and high combined *β*-catenin and DRD1 — H-scores revealed an insignificant correlation (log rank = 2.047; *P* = 0.153).

## Discussion

Colorectal cancer is one of the most prevalent types of cancer worldwide and continuously increasing in incidence [25]. In the era of personalized therapy, the necessity of new target therapy that can improve response to conventional lines of treatment is growing. Not only the discovery of new entities is required but also the innovation of relationships to pathways that play key roles in carcinogenesis in specific tumor types becomes mandatory to potentiate the response to the new therapies. One of the molecules that were only recently detected to play an essential role in colon carcinogenesis and prognosis is DDR1 [22].

Although few studies addressed DDR1 in CRC, all concluded its poor prognostic role [6, 8, 9]. In the present study, we aimed to validate the prognostic role of DDR1 expression in CRC in a random sample of Egyptian patients and to validate its proposed relation to the *Wnt/beta-catenin* pathway which was found to be dysregulated in 90% of CRC cases [26]. Additionally, there was some data suggesting DDR1 could support metastasis across a Wnt/*β*-catenin-dependent mechanism [27].

Regarding the expression of DDR1in the present study, it was found to exhibit a stepwise increase in the studied three groups starting from the adjacent nonneoplastic group to the carcinoma group and across the adenoma group. This assumes that increased expression of DDR1 plays an important role in CRC carcinogenesis. This supports its carcinogenic role in a variety of other human tissues [28]. Indeed, this is not a surprising issue due to the previously reported modulating effect of DDR1 on TGFBI which often acts as a tumor promoter [29]. Additionally, it has been reported that the binding of DDR1 and ECM collagens activates multiple intracellular kinases which in turn trigger several tumorigenic pathways [30] such as JAK-STAT and mTOR signaling pathways. These pathways are among the tumorigenic pathways in CRC [31, 32]. Interestingly, there are some shreds of evidence about how the cross-taking of DDR1 and STAT3 contributes to the development of hepatocellular carcinoma [33]. This adds more substantiation to the possible role of DDR1 in colorectal tumorigenesis.

When DDR1 expression was correlated with available clinicopathologic parameters, this revealed that higher expression levels were evident in higher-grade, higher-stage tumors and significantly correlated to the presence of lymph node metastasis. Although, to the best of our knowledge, our study is the first immunohistochemical study addressing the correlation of DDR1 expression with clinicopathologic parameters, the positive relation to adverse parameters was consistent with previous studies not only regarding CRCs [34] but also in other types of cancer such as renal cell carcinoma [35] and gastric carcinoma [36]. However, no relation has been detected in breast cancer with clinicopathologic prognostic parameters [37]. The adverse effect of increased DDR1 expression in CRC could be explained by its promoting influence on epithelial-to-mesenchymal transition (EMT) [34] in tissue and ligand-dependent manner. This in turn stimulates the progression of malignant tumors and the increase of metastatic optional. Lafitte and his colleagues presumed that DDR1 promotes CRC cells’ migration through Wnt/*β*-catenin-dependent mechanism, RAS-independent mechanism [27], and BCR and PEAK1-dependent mechanism. The latter is phosphorylated by a DDR1 kinase-dependent mechanism [38]. The cells subjected to EMT then acquire the ability to migrate. And in this step, DDR1 was also revealed to play a role in the degradation of extracellular matrix through upregulation of MMP2 that facilitates cancer cell invasion [39]. Therefore, it was not surprising to spot a significant correlation between DDR1 expression and the detection of LN metastasis and a near-significant correlation with distant metastasis in the present study.

Additionally, the impacts of DDR1 overexpression on cells’ proliferation and survival have been reported in several human tissues including few in vitro studies in the colon [28]. For instance, a previous study concluded that colon cancer cells with DDR1 overexpression have the ability to survive more due to the activation of Notch1 which stimulates the expression of pro-survival genes *Hes1* and *Hey2* [40].

Curiously, both H-score, as well as the intensity of DDR1 staining, were inversely correlated to the presence of intratumoral inflammatory response. This finding is just recently tackled in CRC when Duan X and colleagues stated that DDR1 collagen-induced activation inhibits IL-18 synthesis which in turn decreases intratumoral T-cell infiltration. Furthermore, they found that DDR1 increases PDL-1 expression across activation of the c-Jun amino-terminal kinase (JNK) signaling pathway [41]. In contrast, a direct relationship has been recently stated in other types of cancer such as pancreatic carcinoma. This could be due to the activation of collagen-induced DDR1 which in turn stimulates CXCL5 production [42]. The authors of this research focused on neutrophilic response. This explains our contradicting results for ours since they focused on only neutrophils. Therefore, this point is still a matter of debate. Whereas some researchers stated the immune-response induction of DDR1 [42], others stated the opposite [41]. It seems that it depends on the tissue type and the immune component. However, it deserves more investigations to elucidate this relation between DDR1 expression and immune response as it signifies the urgent need to promote target therapy to inhibit DDR1 expression which will not only affect the prognosis of the disease but also may improve the response to emerging immunotherapy.

It is therefore not surprising that DDR1 high expressing group of CRC in our study sample exhibited the shortest overall survival since they demonstrated the most advanced stage, positive LN invasion, and were of the highest histological grade and less intratumoral inflammatory response. This is consistent with a very recent prognostic study addressing DDR1 in CRC [41].

In a trial to discover the intracellular downstream pathways regulated by DDR1 and could be responsible for this adverse outcome in cases overexpressing this protein, we aimed to study its correlation with *β*-catenin which is a key component of the Wnt/*β*-catenin signaling pathway. Dysregulation of the Wnt/*β*-catenin signaling pathway in CRC has been spotted in several studies with an adverse impact on prognosis [43, 44]. Curiously, we spotted a significant association between the expression of both proteins. To the best of our knowledge, it is the first study focusing on the immune expression of both proteins in the same cohort of patients. *β*-catenin is an intracellular protein that poses a close association with E-cadherin which maintains epithelial cell adhesion, and its abrogation would promote epithelial to mesenchymal transition (EMT). Since DDR1 is a collagen receptor that regulates cell adhesion, it is very likely to have a relationship with E-cadherin/*β*-catenin system. Thus, *β*-catenin has an indirect effect on the progression of some carcinomas [45]. This is consistent with results reported by Jietany et al. who suggested that DDR1-mediated phosphorylation of BCR is necessary for maintaining the transcriptional activity of *β*-catenin which is important for invasion by tumor cells [38]. Additionally, this is in accordance with data about the role of mutations in APC complex components and *β*-catenin which assists the accumulation of cytoplasmic *β*-catenin leading to its translocation to the nucleus [46]. This sequentially increases the level of nuclear *ß*-catenin in the cytoplasm which behaves as a transcription factor that leads to the activation of a number of target genes such as CyclinD1 and cMyc that stimulate uncontrolled tumor cells’ proliferation [47]. Some studies reported promoting the effect of DDR1 on cell proliferation, motility, and invasion, relying on the type of tumor and surrounding microenvironment [48, 49]. Thus, targeting both DDR1 and *β*-catenin could improve the prognosis of CRC cases.

In conclusion, DDR1 overexpression is a frequent feature in CRC as well as CR adenoma. Overexpression of DDR1 in CRC is a poor prognostic factor and a suppressor of the antitumor immune response. DDR1 and *β*-*catenin* proteins seem to have a synergistic action that needs more extensive investigations. Interestingly, this study shed the light on the important therapeutic implication of DDR1 inhibitors which could modulate the prognosis of CRC in a subset of patients and improve response to immune therapy.

## Supplementary Information

**Additional file 1.** Sample size estimation.

## Data Availability

The datasets supporting the conclusions of this article are included within the article.
