# Supplementary material for: Clinical significance of immunohistochemical expression of DDR1 and β-catenin in colorectal carcinoma
Source: World J Surg Oncol. 2023 Jun 5;21:168. doi: 10.1186/s12957-023-03041-6 (PMC10240746; doi:10.1186/s12957-023-03041-6)
Supplement: Supplementary file 1 — Additional file 1. Sample size estimation. [file 12957_2023_3041_MOESM1_ESM.pdf]

Academic research department

Public health and community medicine

Department

Menoufia Faculty of Medicine
Accredited

Sample size estimation

Name: Marwa Mohammed Dawoud, Marwa Salah, Asmaa Shams El Dein Mohamed.

Title of the study: Clinical Significance of Immunohistochemical Expression of DDR1 and β -Catenin in Colorectal Carcinoma.

Degree: Scientific research.

Study design: Retrospective study.

Sample size estimation

It's a retrospective study aiming to investigate the immunohistochemical expression of DDR1 and β -Catenin in colorectal carcinoma and its correlation with clinicopathologic data. Data will be collected from all surgically resected tissue specimens of CRC cases which are available at the archives of Pathology Department, Faculty of medicine, Menoufia University between Jan 2018 and Dec 2019.

Sample size: 76 colon specimens including 48 CRC cases, 20 cases of colorectal adenoma, and 8 control cases (non-neoplastic tissue adjacent to carcinomatous tissue).

Dr.Angham Solaiman
